# An unusual case of simultaneous left glomus vagale, jugulare and tympanicum tumor

**DOI:** 10.11604/pamj.2017.26.241.11760

**Published:** 2017-04-27

**Authors:** Salah Bellasri, Mounir Hmidi

**Affiliations:** 1 Medical Imaging Department, Military Hospital, University Mohammed V, Rabat, Morocco; 2 Department of Otorhinolaryngology-Head and Neck Surgery, Military Hospital Meknes, Morocco

**Keywords:** Glomus jugulare, glomus tympanicum, glomus vagale, chemodectoma, paraganglioma

## Image in medicine

A 49-year-old male who attended our hospital due to a history of left hearing loss over a period of 2 years, associated to otalgia, and vertigo to which bleeding in the left ear and facial paralysis had been added in the last 4 months. He reported the simultaneous onset of a left-sided neck swelling with slow growing. After this, he developed loss of sensation of taste on the posterior third of the tongue and swallowing dysfunction, dysphonia, atrophy of the trapezius and sternocleidomastoid muscle and deviation and atrophy of tongue toward the affected side. On physical examination, the mass was found to be painless, soft, pulsating and semifixed. During the otoscopic examination, a pulsatile erythematous lesion of vascular appearance was noted in the external auditory canal. The audiometric test revealed severe left mixed hearing loss. A Doppler ultrasound was performed, which revealed a hypervascularized tumor in the left-sided neck that extended to the skull base. An axial and coronal computerized tomography (CT) scan was taken, revealing a soft tissue lesion extending from the border of II and III neck region to the middle ear and external auditory canal, causing large jugular bulb, hypoglossal canal and temporal bone erosion. This mass avidly enhanced after CT angiography. Following the results of preoperative examinations, diagnosis of simultaneous left glomus vagale, jugulare and tympanicum was confirmed. As the tumor was unresectable, the patient received fractionated radiotherapy associated with iterative stereotactic radio surgery. The patient has been stable over the last two years.

**Figure 1:**
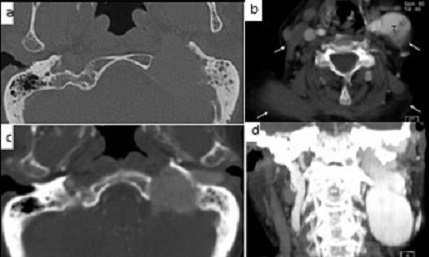
A) computerized tomography, axial image demonstrates large extensive bone erosion and destruction of temporal and jugular bulb; B) post contrast CT angiography (CTA) axial image revealed a solid, well defined mass that seemed to be a highly vascularized tumor: notable atrophy of the trapezius and sternocleidomastoid muscle (white arrows), which indicate invasion of the left cranial nerve XI; C) CTA showed an important enhancement with extension on periauricular region; D) coronal CTA thick image demonstrated the extension in height of the tumor

